# Higher systemic immune-inflammation index is associated with sarcopenia in individuals aged 18–59 years: a population-based study

**DOI:** 10.1038/s41598-023-49658-1

**Published:** 2023-12-13

**Authors:** Jinlong Zhao, Lingfeng Zeng, Guihong Liang, Yaoxing Dou, Guanghui Zhou, Jianke Pan, Weiyi Yang, Kunhao Hong, Jun Liu, Li Zhao

**Affiliations:** 1 The Second Clinical College/State Key Laboratory of Traditional Chinese Medicine Syndrome of Guangzhou University of Chinese Medicine, Guangzhou, 510405 China; 2 The Second Affiliated Hospital of Guangzhou University of Chinese Medicine (Guangdong Provincial Hospital of Chinese Medicine), Guangzhou, 510120 China; 3 The Fifth Clinical College of Guangzhou University of Chinese Medicine, No.12, Jichang Road, Baiyun District, Guangzhou City, 510405 China; 4 The Research Team on Bone and Joint Degeneration and Injury of Guangdong Provincial Academy of Chinese Medical Sciences, Guangzhou, 510120 China; 5 Guangdong Second Chinese Medicine Hospital (Guangdong Province Engineering Technology Research Institute of Traditional Chinese Medicine), Guangzhou, 510095 China; 6 Guangdong Provincial Hospital of Chinese Medicine, No.53, Jingle Road, Xiangzhou District, Zhuhai, 519015 Guangdong Province China

**Keywords:** Immunology, Medical research

## Abstract

The association between the systemic immune-inflammation index (SII) and the risk of sarcopenia has not yet been revealed. The purpose of this study was to investigate the relationship between the SII and sarcopenia in individuals aged 18–59 years. All data for this study are from the National Health and Nutrition Examination Survey (NHANES) database, including 7258 participants (age range: 18–59 years). We divided SII values by quartiles (quartiles 1–4: 0.3–3.1, 3.2–4.4, 4.4–6.2, and 6.2–58.5). We constructed a multivariate logistic regression model to assess the association between the SII and the risk of sarcopenia, and an interaction test was run to test the stability of the model and identify high-risk individuals with sarcopenia. Compared to nonsarcopenia participants, sarcopenia patients had a significantly higher SII value (weighted average: 6.65 vs. 5.16) (*P* = 0.002). Multivariate logistic regression results showed a positive linear relationship between the SII and sarcopenia (OR [odds ratio] = 1.12, 95% CI [confidence interval] 1.03–1.21). Compared to the quartile 1 group, the quartile 4 group was associated with a higher risk of sarcopenia (OR = 3.94, 95% CI 1.42–10.94). Compared with the quartile 1 group, the OR value of the quartile 2 to quartile 4 groups showed an upwards trend (*P*_trend_ < 0.001) as the level of SII increased. Subgroup analysis also indicate that the correlation between higher SII values and the risk of sarcopenia was stable. There was a significant positive linear relationship between SII and sarcopenia, indicating that higher SII values can increase the risk of sarcopenia in individuals aged 18–59 in the United States. The findings of this study will be beneficial in promoting the use of SII alone or in combination with other tools for the risk screening of sarcopenia in communities or large populations.

## Introduction

Sarcopenia is a progressive, systemic skeletal muscle disease^1,2^ characterized by decreased body function, muscle strength, and mass. Due to differences in the definition, evaluation tools, and diagnostic cut-off values of sarcopenia, the prevalence of sarcopenia varies greatly in different countries or regions^3,4^. In the past few decades, the prevalence of sarcopenia has been increasing, with over 10–16% of the general population aged 60 and over suffering from sarcopenia^3,5^. Epidemiological statistical results show that the prevalence rates of sarcopenia in the community, hospitalized, and care-home populations are 10%, 23%, and 38%, respectively^6^. The prevalence of sarcopenia in men and women is 14% and 12%, respectively^6^. Studies have shown that sarcopenia is highly correlated with cachexia^7–10^ and increases the risk of falls and fractures. In addition, sarcopenia is also believed to be associated with activity disorders, low bone mass, metabolic disorders, depression, and hospital mortality^11–15^, which undoubtedly reduces the quality of life of patients and increases the medical burden. In view of the enormous harm of sarcopenia to public health, it is of important clinical and public health value to identify and screen high-risk populations with sarcopenia early in the community and provide targeted prevention and treatment recommendations.

Currently, there is still no international consensus on the diagnostic methods and standards for sarcopenia. The commonly used diagnostic criteria were proposed by working groups such as the European Working Group on Sarcopenia in Older People (EWGSOP)^16,17^, the International Working Group on Sarcopenia (IWGS)^18^, the Asian Working Group for Sarcopenia (AWGS)^1^, and the Foundation for the National Institutes (FNIH)^19^. However, the vast majority of criteria recommend that sarcopenia be defined based on low muscle content and/or low muscle strength and/or low physical fitness^15–19^. The measurement of muscle mass relies on instruments such as CT, MR, and dual-energy X-ray absorptiometry (DXA), which is not convenient for use in large-scale population screening. In recent years, different scales have been widely used in population screening due to their rapid and simple advantages, including the sarcopenia-five scale (SARC-F)^20^, SARC-F combined with calf circumference (SARC-CalF) scale^21^, and SARC-F adding elderly and body mass index (BMI) information scale (SARC-F + EBM)^22,23^, but those scales have difficulty taking into account the sensitivity and specificity of the diagnosis of sarcopenia. Therefore, the efficacy of those scales in the diagnosis or screening of sarcopenia still needs to be further verified. In this context, simple and efficient screening tools or evaluation indicators for sarcopenia need to be explored and developed.

The systemic immune-inflammation index (SII) is an index for evaluating inflammation based on neutrophil, lymphocyte, and platelet counts^24,25^, which can more objectively reflect changes in the level of inflammation in the body. SII can be calculated through a routine blood examination, and it has the advantages of being fast, efficient, simple, and low in cost. Previous studies have confirmed that the SII has good clinical application value in the diagnosis of chronic diseases such as tumours, cardiovascular diseases, osteoporosis, and rheumatoid arthritis^26–29^. Research suggests that chronic low-grade inflammation associated with ageing may be an important cause of sarcopenia^30^. With age, the body presents a chronic inflammatory state^30^. The levels of various inflammatory substances and proinflammatory factors increase, promote protein decomposition and inhibit protein synthesis through various signalling pathways^31–33^, resulting in an imbalance in muscle tissue synthesis and metabolism. Shi et al.^34^ found that higher levels of SII were associated with an increased risk of low muscle mass in a large population, and they pointed out that anti-inflammatory therapy may be a possible pathway to alleviate low muscle mass status. Another study showed that NLRP3 inflammasomes and cell death can lead to muscle dysfunction by reducing glycolytic potential and muscle fibre size^35^. In addition, Karanth et al.^36^ analysed cross-sectional data and concluded that measures need to be taken to prevent sarcopenia in populations with high levels of systemic inflammation. Based on the causal relationship between inflammation levels and sarcopenia, the SII may have clinical application value in predicting the risk of sarcopenia. However, there is currently a lack of research to confirm this conjecture. Our hypothesis is that there is a potential correlation between the SII and sarcopenia. In addition, using the SII for a simple risk assessment of 18–59-year-olds has better value, which may help us identify susceptible populations earlier and take more preventive measures. To fill this gap, we used population data from the National Health and Nutrition Examination Survey (NHANES) database to investigate the relationship between the SII and sarcopenia, which will provide new measurement tools and options for community or large-scale population screening for sarcopenia.

## Methods

### Data sources and study participants

The data for this study were obtained from the NHANES database. NHANES is a cross-sectional survey conducted by the Centers for Disease Control and Prevention (CDC) of the United States^37^ and is aimed at collecting health and nutrition information for the general population in the United States. The survey participants of NHANES are determined through stratified cluster multistage sampling, which can be used as a representative sample of American residents. Since the independent variable and end point outcome of this study are SII and sarcopenia, respectively, we only selected two survey periods, 2011–2012 and 2013–2014, with sufficient measurement data to calculate those two variables. All participants signed an informed consent form^38^; NHANES was approved by the Ethics Review Committee of the National Center for Health Statistics (Protocol # 2011–17)^38^.

**Figure 1:**
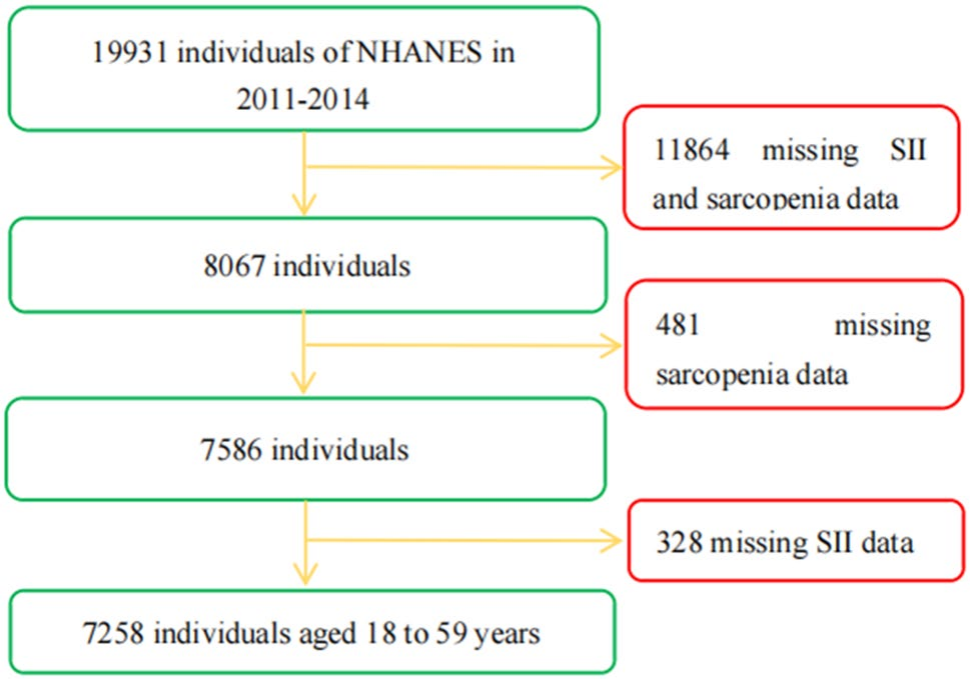
Flow chart of study participants.

A total of 19,931 participants were included in the 2011–2014 survey cycle. Since SII- and sarcopenia-related data are the core data of this study, all participants lacking data on those two variables were not included for further analysis. Specifically, participants who were only missing covariate data were not excluded, and those missing values were set as dummy variables in the statistical analysis. There were no other exclusion or inclusion criteria. A total of 7258 participants were eventually included. The inclusion and exclusion process is shown in Fig. 1.

### Determination of sarcopenia outcomes

According to FNIH sarcopenia project criteria (FNIH)^19,39^, we will determine whether participants in the 2011–2014 cycle have health issues with sarcopenia based on data from the NHANES database. According to the FNIH standard, the judgement factors for sarcopenia include weakness and muscle loss^19^. The quantitative judgement indicators for these two factors are grip strength and appendicular skeletal muscle mass (ASM). Both grip strength and ASM require BMI adjustment^19^, and the adjusted grip strength and ASM are named grip strength_BMI_ and ASM_BMI_, respectively. In this study, the cut-off values of grip strength_BMI_ for males and females were < 1.0 and < 0.56^19^, respectively. The ASM_BMI_-cutoff values for males and females were < 0.789 and < 0.512, respectively^19^. Based on the diagnostic criteria and cutoff values of FNIH, we can diagnose whether each participant has sarcopenia based on their grip strength_BMI_ and ASM_BMI_ data.

During the 2011–2014 cycle, body composition (including muscle mass) was measured using DXA, and all results were calculated using HologicQDR-4500 software (version Apex 3.2, Hologic, USA)^40,41^. Participants were asked not to undergo other radiological examinations the day before or on the day of the DXA evaluation. Specific information and data on body composition measurements can be found on the CDC website^40,41^. The grip strength test was conducted using a Takei dynamometer (TKK 5401, Takei Scientific Instruments, Tokyo, Japan), and measurement details can be found on the CDC website and measurement manual^42,43^. The measurement work related to clinical and laboratory indicators is carried out by trained medical workers. In addition, the detailed process of sample collection and processing has been described in detail in the NHANES Laboratory/Medical Technician Procedure Manual (LPM)^40–43^.

### Measurement of SII

The SII is an immune inflammatory index calculated based on the counts of neutrophils, platelets, and lymphocytes in peripheral blood. The calculation formula is SII = platelet count × neutrophil count/lymphocyte count^44^. Blood sample collection for all participants was completed at the NHANES Mobile Examination Center (MEC). The instruments used for the analysis of whole blood cell counts in the 2011–2012 and 2013–2014 cycles were the Coulter HMX (Coulter Electronics Ltd., Bedfordshire, UK)^45^ and Beckman Coulter DXH 800 (Beckman Coulter, Brea, CA, USA)^46^, respectively.

### Assessment of covariates

The selection of covariates in this study was mainly based on the literature and variables recognized by academia^47–51^, and they primarily consisted of sociodemographic factors. The covariates mainly included age (years), sex, race (non-Hispanic white, non-Hispanic black, American Mexican, and others), BMI, and smoking status (never, former, current). Based on BMI values, we divided BMI into three groups: < 25, 25–24.99, and ≥ 30. In addition, we extracted six diseases, including diabetes^52^, chronic kidney disease (CKD)^53^, hypertension^54^, chronic obstructive pulmonary disease (COPD)^55^, coronary heart disease (CHD)^56^ and hyperlipidaemia^57^, as covariates. The diagnosis of these diseases was based on clear diagnostic criteria^52–57^ and matched the measurement indicators in the NHANES database for diagnosis; or, each participant included in this study reported taking disease-related drugs, such as hypoglycaemic drugs, lipid-lowering drugs, etc.; or, participants reported having been diagnosed by a doctor.

### Statistical analyses

The data analysis of this study was conducted in accordance with CDC statistical guidelines^58^. All data extraction and analysis were performed using R software (version 4.2.2, http://www.R-project.org, The R Foundation) and EmpowerStats software (version 4.1, www.empowerstats.com, X&Y solutions, Inc. Boston, MA, USA). All tests were bilateral and had the following test level: α = 0.05. Continuous variables were represented by weighted means and 95% confidence intervals (CIs); classification variables were expressed using frequency and weighted percentages (%). We used a bivariate logistic regression model to evaluate the relationship between the SII and sarcopenia. Due to the large absolute value of SII and to make the data have higher statistical efficiency and facilitate clinical application, we divided all SII values by 100 as the original data. Therefore, in this study, each unit increase in SII means an increase of 100 in the absolute value of SII. To test the stability of the impact of SII on sarcopenia in different regression models, we divided SII values into four groups according to the quartile. The quartile ranges of SII were 0.3–3.1 (quartile 1), 3.2–4.4 (quartile 2), 4.4–6.2 (quartile 3), and 6.2–58.5 (quartile 4). SII was entered into the model as a continuous variable and as a categorical variable (quartile grouping). To further test the stability of the relationship between SII and sarcopenia, we constructed three regression models to gradually adjust for confounding factors: Model 1 did not adjust for covariates; Model 2 adjusted for age, sex, BMI, and ethnicity; and based on Model 2, Model 3 further adjusted for smoking status, diabetes, CKD, hypertension, COPD, CHD, and hyperlipidaemia.

We further conducted interaction tests (subgroup analysis) on these covariates to help us evaluate the reliability of the conclusions of this study from a comprehensive perspective. Considering the multiple comparisons in subgroup analysis, we adjusted the *P*-value using Bonferroni correction to obtain a conservative threshold for determining significant differences (*P* = 0.05/8 = 0.006).

### Ethics approval and consent to participate

The protocols of NHANES were approved by the institutional review board of the National Center for Health Statistics, CDC (https://www.cdc.gov/nchs/nhanes/irba98.htm). NHANES has obtained written informed consent from all participants.

## Results

### Baseline characteristics of the study participants

A total of 7258 participants were included in this study, including 88 sarcopenia patients and 7170 nonsarcopenia participants. The age range for all participants was 18–59 years, with a weighted average age of 38.5 years. The weighted average ages of participants with sarcopenia and nonsarcopenia were 45.39 and 38.45, respectively, with a statistically significant difference (*P* = 0.0003). The weighted average values (and 95% CI) of SII in sarcopenia and nonsarcopenia participants were 6.65 (95% CI 5.79–7.52) and 5.16 (95% CI 5.03–5.30), respectively, with a statistically significant difference (*P* = 0.002). The weighted average ages of the quartile 1–4 groups were 37.5, 38.12, 39.32, and 39.07, respectively, with statistically significant differences between the groups (*P* = 0.022). The frequency of sarcopenia in the quartile 1–4 groups was 11, 17, 21, and 39, respectively, with a statistically significant difference between the groups (*P* = 0.0005). The weighted average (and 95% CI) SII values in the quartile 1–4 groups were 2.42 (95% CI 2.40–2.46), 3.81 (95% CI 3.79–3.83), 5.25 (95% CI 5.22–5.27), and 8.84 (95% CI 8.67–9.01), respectively, with statistically significant differences between the groups (*P* < 0.001). The specific characteristics of all 7258 participants are shown in Table 1.

**Table 1:** Characteristics of individuals by SII quartile from NHANES, 2011–2014.

Characteristic	SII levels (N = 7258)				
	Quartile 1 (< 3.1)	Quartile 2 (3.2–4.4)	Quartile 3 (4.4–6.2)	Quartile 4 (> 6.2)	*P-*Value
Age, years					0.033
< 40	1026 (55.58)	996 (53.27)	959 (48.09)	964 (49.18)	
40–49	374 (20.88)	429 (23.09)	447 (26.54)	442 (26.02)	
≥ 50	415 (23.54)	389 (23.64)	408 (25.37)	409 (24.79)	
Sex					< 0.001
Male	1050 (58.96)	921 (51.48)	874 (49.82)	724 (40.38)	
Female	765 (41.04)	893 (48.52)	940 (50.18)	1091 (59.62)	
Race/ethnicity					< 0.001
Non-Hispanic White	500 (54.28)	693 (63.92)	764 (66.74)	772 (66.10)	
Non-Hispanic Black	634 (20.17)	381 (10.87)	314 (8.69)	314 (8.86)	
American Mexican	213 (10.27)	221 (9.04)	256 (9.98)	251 (10.27)	
Others	468 (15.28)	519 (16.18)	480 (14.60)	478 (14.78)	
BMI					< 0.001
< 25	733 (38.30)	656 (36.72)	561 (28.39)	536 (27.51)	
25.0–29.9	546 (32.13)	589 (33.68)	568 (32.67)	498 (29.60)	
≥ 30	536 (29.57)	569 (29.60)	685 (38.94)	781 (42.89)	
Smoking status					0.053
Never	1070 (58.33)	1090 (58.34)	1077 (58.66)	1020 (55.88)	
Current	394 (21.42)	363 (20.61)	407 (21.08)	448 (24.30)	
Former	262 (16.73)	290 (18.74)	279 (18.30)	290 (18.28)	
Missing	89 (3.52)	71 (2.31)	51 (1.96)	57 (1.55)	
Diabetes					< 0.001
No	1545 (86.37)	1546 (87.83)	1486 (82.54)	1389 (78.88)	
Yes	152 (6.94)	165 (6.68)	183 (9.57)	235 (10.54)	
IFG or IGT	109 (6.21)	97 (5.16)	123 (6.73)	128 (7.22)	
Missing	9 (0.49)	6 (0.34)	63 (1.16)	63 (3.36)	
CKD					0.0001
No	1630 (91.30)	1646 (91.96)	1614 (89.79)	1545 (86.74)	
Yes	146 (6.82)	134 (6.59)	160 (8.06)	236 (11.81)	
Missing	39 (1.89)	34 (1.46)	40 (2.15)	34 (1.45)	
Hypertension					0.002
No	1374 (76.68)	1359 (75.42)	1302 (71.14)	1274 (69.73)	
Yes	441 (23.32)	455 (24.58)	512 (28.86)	541 (30.27)	
COPD					0.22
No	1635 (92.22)	1632 (93.10)	1663 (93.25)	1636 (92.46)	
Yes	26 (2.20)	36 (2.15)	40 (2.95)	56 (3.56)	
Missing	254 (5.58)	146 (4.75)	111 (3.80)	123 (3.98)	
CHD					0.418
No	1638 (93.04)	1651 (94.35)	1681 (94.71)	1666 (94.88)	
Yes	16 (0.98)	15 (0.75)	15 (1.05)	22 (1.05)	
Missing	161 (5.98)	148 (4.90)	118 (4.24)	127 (4.07)	
Hyperlipidemia					< 0.001
No	791 (41.42)	700 (38.59)	653 (33.07)	599 (32.49)	
Yes	1024 (58.58)	1114 (61.41)	1161 (66.93)	1216 (67.51)	
Number of sarcopenia					0.0005
No	1804 (99.47)	1797 (99.12)	1793 (98.52)	1776 (97.41)	
Yes	11 (0.53)	17 (0.88)	21 (1.48)	39 (2.59)	
SII	2.42 (2.40, 2.46)	3.81 (3.79, 3.83)	5.25 (5.22, 5.27)	8.84 (8.67, 9.01)	< 0.001

*N* Number of observed; *SII* Systemic immune-inflammation index; *BMI* Body mass index; *IFG* Impaired fasting glucose; *IGT* Impaired glucose tolerance; *CKD* Chronic kidney disease; *COPD* Chronic obstructive pulmonary disease; *CHD* Coronary heart disease.

For continuous variables: survey-weighted mean (95% CI), *P*-value was by survey-weighted linear regression.

For categorical variables: N (survey-weighted percentage, %), *P*-value was by survey-weighted Chi-square test.

### Association between SII and sarcopenia

We constructed three logistic regression models to explore the independent impact of SII on sarcopenia. The unadjusted Model 1 results showed that an increase in SII was associated with a higher risk of sarcopenia (OR [odds ratio] = 1.10, 95% CI 1.05–1.15). Both the microadjustment model (Model 2) and the overall covariate adjustment model (Model 3) showed that a higher SII is associated with a higher risk of sarcopenia (Table 2), and their corresponding effect values (and 95% CI) were OR = 1.14 (95% CI 1.07–1.21) and OR = 1.12 (95% CI 1.03–1.21), respectively, indicating a positive linear relationship between SII and sarcopenia. In Model 1 (OR = 4.97, 95% CI 2.37–10.42), Model 2 (OR = 4.53, 95% CI 2.06–9.93) and Model 3 (OR = 3.94, 95% CI 1.42–10.94), the quartile 4 group was associated with a higher risk of sarcopenia than the quartile 1 group. Compared with the quartile 1 group, the OR value of the quartile 2–4 groups showed an upwards trend (*P*_trend_ < 0.001) as the level of SII increased. The above univariate and multivariate logistic regression analysis results indicate that there is a potential positive correlation between SII values and the risk of sarcopenia.

**Table 2:** Association of SII with sarcopenia in 7, 258 participants aged 18–59 years.

Exposure	Model 1	*P*-value	Model 2	*P*-value	Model 3	*P*-value
	OR (95% CI)		OR (95% CI)		OR (95% CI)	
SII	1.10 (1.05, 1.15)	< 0.001	1.14 (1.07, 1.21)	< 0.001	1.12 (1.03, 1.21)	0.009
Stratifed by SII quartiles						
Quartile 1	Reference		Reference		Reference	
Quartile 2	1.66 (0.70, 3.93)	0.26	1.88 (0.79, 4.43)	0.166	1.58 (0.55, 4.53)	0.322
Quartile 3	2.81 (1.18, 6.70)	0.027	2.25 (0.90, 5.61)	0.096	1.93 (0.59, 6.35)	0.217
Quartile 4	4.97 (2.37, 10.42)	< 0.001	4.53 (2.06, 9.93)	0.001	3.94 (1.42, 10.94)	0.013
*P* for trend	0.0002		0.0002		< 0.001	

Model 1: no covariates were adjusted.

Model 2: age, sex, BMI, and race/ethnicity were adjusted.

Model 3: age, sex, BMI, race/ethnicity, smoking status, diabetes, CKD, hypertension, COPD, CHD, and hyperlipidemia were adjusted.

### Subgroup analysis

**Figure 2:**
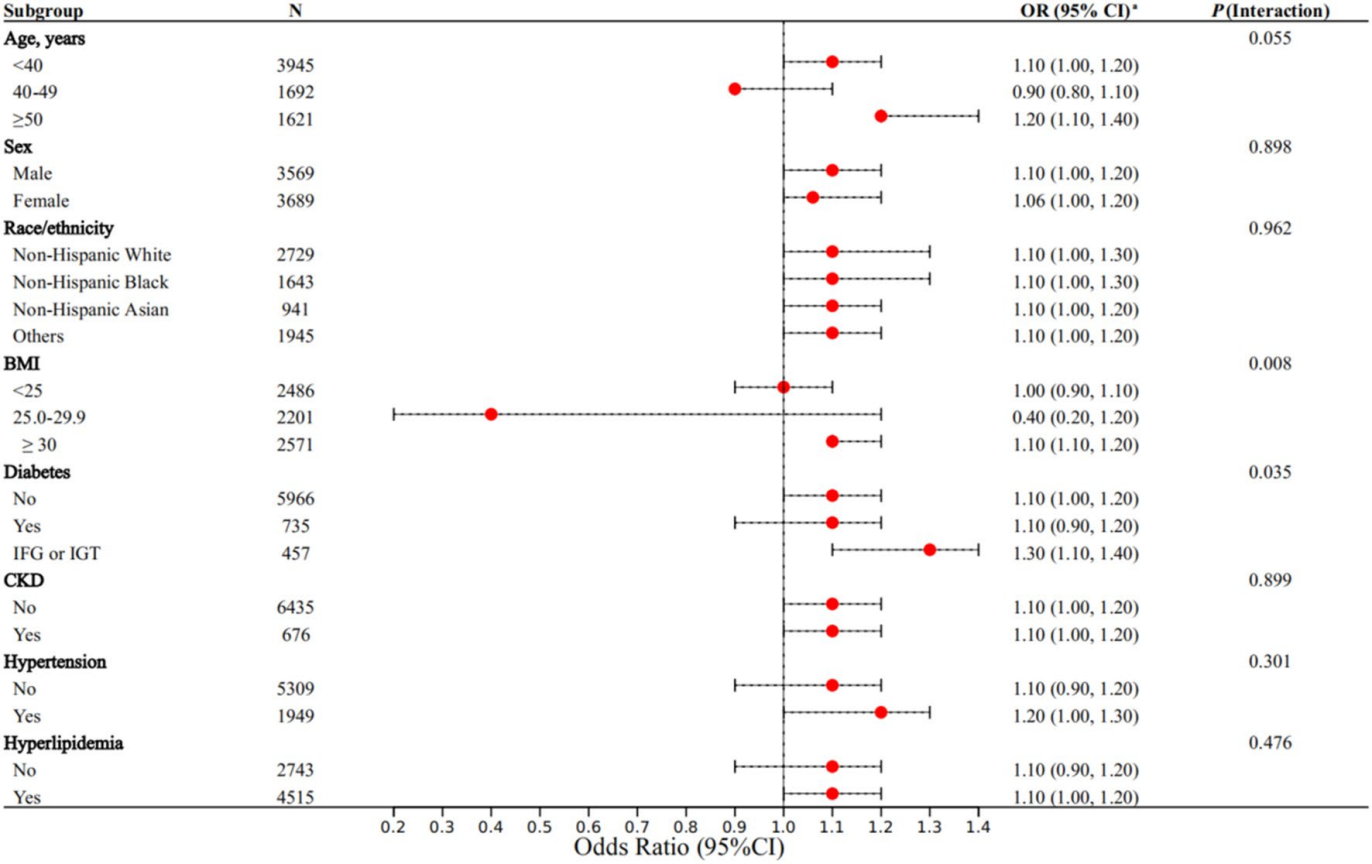
Forest plots of subgroup analysis for the association between SII and sarcopenia. Age, sex, BMI, race/ethnicity, smoking status, diabetes, CKD, hypertension, COPD, CHD, and hyperlipidemia were all adjusted except the variable itself. N: Number of observed. (**a**) Survey-weighted OR (95% CI). P-interaction: by global Chi-square test for interaction terms.

To further test the reliability of this regression model, we conducted a subgroup analysis of covariates (Fig. 2). We found that higher SII values are associated with the risk of sarcopenia, a conclusion that is highly similar across age, sex, race, CKD, hypertension, diabetes, BMI, and hyperlipidaemia subgroups (all *P* for interaction > 0.006) (Fig. 2).

## Discussion

Sarcopenia is believed to be closely and positively correlated with cachexia, decreased physiological function, and increased mortality^59,60^, which makes it necessary to pay attention to the screening, diagnosis, prevention, and treatment of sarcopenia. Considering the enormous harm of sarcopenia to public health, early identification of high-risk populations with sarcopenia and recommendations for prevention or treatment may be the most effective way to reduce the harm of sarcopenia to public health. Therefore, it is an important task to find an evaluation tool or indicator for sarcopenia suitable for large-scale population screening to achieve this goal. To the best of our knowledge, this study is the first to explore the correlation between SII and the risk of sarcopenia, which will provide reliable data and more options for community screening among people at risk for sarcopenia. This study revealed a positive linear relationship between SII and the occurrence of sarcopenia (OR = 1.12, 95% CI 1.03–1.21, P = 0.009), indicating that an increase in SII will significantly increase the risk of sarcopenia. In addition, compared to the quartile 1 group (SII: 0.3–3.1), the quartile 4 group (SII: > 6.2) had a significantly increased risk of sarcopenia by 294% (OR = 3.94, 95% CI 1.42–10.94, *P*_trend_ < 0.001). The interaction tests indicate that the correlation between higher SII values and the risk of sarcopenia is not affected by age, sex, race, CKD, hypertension, diabetes, BMI, and hyperlipidaemia. The above three stable conclusions suggest that higher SII levels are associated with a higher risk of sarcopenia. In addition, there is believed to be a causal relationship between inflammation and sarcopenia, and studies have pointed out that inflammation-related factors can be used as therapeutic targets for sarcopenia^35,61,62^. We are very encouraged by these conclusions because they indicate that an objective, easy-to-operate, and scalable SII has great potential to become a risk assessment indicator for sarcopenia that can be used in community or large-scale population screening.

The SII is a comprehensive inflammatory index that reflects the immune and inflammatory status of the host and is calculated by measuring neutrophils, lymphocytes, and platelets in the peripheral blood^63^. Therefore, the data acquisition and calculation of SII has the advantages of accuracy, objectivity, efficiency, and ease of promotion. The increase in SII values reflects an increase in neutrophil and platelet counts, as well as an increase in multiple cytokine levels (and/or a decrease in lymphocyte counts). The decrease in lymphocytes during inflammation can increase the production of proinflammatory cytokines, induce oxidative stress, and promote cell apoptosis^64^, thereby aggregating inflammation to promote the development of disease. Ageing is often accompanied by an increase in inflammatory markers and related factors, and sarcopenia is also considered to be an age-related disease^30^. Significantly elevated levels of inflammation and oxidative stress are believed to be important factors inducing sarcopenia in middle-aged and elderly people^30,65^. Inflammatory cytokines may antagonize the anabolism of insulin-like growth factor-1 (IGF-1)^66^, reducing the level of muscle IGF-1, which can lead to increased protein catabolism and muscle anabolism disorders. With age, circulating levels of inflammatory markers such as interleukin-6 (IL-6), C-reactive protein (CRP), tumour necrosis factor α (TNF-α), and IL-1β significantly increase and are directly related to decreased muscle mass and strength^67–69^. IL-6 is involved in the regulation of myoprotein turnover and is considered to be a catabolic cytokine^70,71^. Inflammation can also indirectly reduce the concentration of growth hormone (GH) and IGF-1 in the body, which can have a negative impact on skeletal muscle^72,73^, inducing the occurrence of sarcopenia. TNF-α inhibits the synthesis of muscle proteins by regulating the PI3K/Akt/mTOR signalling pathway and promotes muscle atrophy by activating the expression of a series of muscle growth inhibitory factors, such as atrogin-1, nuclear factor kappa (NF-κB), and myostatin^74,75^. In addition, many inflammatory factors (such as IL-15 and CRP) or inflammatory signalling pathways (e.g., MAPK/NF-κB/Wnt/mTOR signalling pathways) are believed to be associated with the occurrence of sarcopenia^76–80^. Based on the above information, we believe that the SII has a solid physiological and pathological basis in predicting the occurrence of sarcopenia, and this is also reflected in our research conclusions. Therefore, SII as a biomarker for sarcopenia warrants further research. Our research findings have increased the understanding of the relationship between the SII and sarcopenia. In the US population aged 18–59 years, SII values may be used for screening individuals at risk for sarcopenia. Moreover, anti-inflammatory therapy may be a potential pathway for the treatment of sarcopenia.

Early identification of high-risk individuals with sarcopenia in specific populations can help the public health system or clinical subspecialties provide more accurate healthcare advice. The results of subgroup analysis showed that in the population with IFG (or IGT) (OR = 1.3), BMI ≥ 30 (OR = 1.1), or age 50–59 years (OR = 1.2), each unit increase in SII (absolute value increase of 100) increased their risk of sarcopenia by 30%, 10%, and 20%, respectively. Research on the relationship between IFG (or IGT) and sarcopenia is still lacking, and our research provides the most basic addition to this topic. Previous studies have shown that serum levels of various inflammatory factors, including IL-6, TNF-α, CRP, plasminogen activator inhibitor-1, and fibrinogen, in IFG (or IGT) populations are significantly higher than those in healthy individuals^81,82^. Moreover, IGT patients generally have more insulin resistance^83^, which is often a chronic inflammatory reaction, compared to healthy people. The IFG population has also been considered to be in a low-inflammatory microenvironment for a long time^84^. Chronic persistence and low-inflammatory status are the main characteristics of obesity^85^ and are also important causes for insulin resistance. Research suggests that obesity is associated with an increase in the expression of inflammatory mediators in adipose tissue which disrupt metabolic homeostasis, and obese individuals are in a chronic inflammatory state of imbalance between proinflammatory and anti-inflammatory immune cells^86,87^. Previous studies have confirmed the association between inflammation and sarcopenia^30,33,67,68^. Seung Jae Heo et al. found that the risk of sarcopenia increased significantly with every 5 consecutive years of age increase^88^; our study showed that the risk of sarcopenia increased more significantly with the increase of SII values in the population aged 50–59 years. Yoo et al. found that chronic inflammatory status in the elderly can cause inflammatory cytokines to antagonize the synthesis and metabolism of IGF-1, leading to muscle synthesis disorders^63^.

### Strengths and limitations

The topic of this study has obvious advantages, which will be beneficial to promoting the screening of individuals at risk for sarcopenia in the community. Prior to this study, there were no studies exploring the relationship between SII and sarcopenia. This study is the first to explore the relationship between SII and the risk of sarcopenia and confirms a linear positive correlation between the two. Second, the participants included in this study were between the ages of 18–59, and conducting risk screening for sarcopenia in this age group (not elderly individuals) can yield better screening and prevention value. Our research conclusions are based on survey data from people aged 18–59, which means that the conclusions of this study have better applicability to the target population. SII does not rely on complex instruments but can be calculated with routine blood tests and has objectivity and repeatability, which will be very beneficial for community doctors or large-scale population screening applications. Considering the enormous public health hazards of sarcopenia, we believe that SII can be used alone or in combination with other tools (questionnaires) to increase the efficiency and accuracy of screening for sarcopenia.

However, we must admit that this study also has the following limitations. First, this was an observational study, which means that the determination of causal relationships still requires further development in the future, requiring high-quality cohort studies, metabolomics studies, or Mendelian randomization studies. Second, due to the lack of diagnostic criteria for sarcopenia in the 18–59 age group, we applied FNIH sarcopenia project criteria to define sarcopenia in this study. And the major limitation is that the criteria were developed mainly for use in older adults. Further development of diagnostic criteria for sarcopenia in the 18–59-year-old population is needed to further test the conclusions of this study. Third, there were differences in the timing of DXA evaluation and blood sample collection for each participant, which may affect the homogeneity of measurement results. Therefore, we need to consider the impact of the accuracy of the measurement results on the reliability of this conclusion. Fourth, some key covariates (e.g., physical activity level and dietary habits) should be included in the analysis as they may have a critical impact on sarcopenia. However, due to the lack of raw data and the difficulty in collecting and quantifying some variables (such as dietary habits), we were unable to include that information. Last, the target population of this study is based on the United States population, which may make the conclusions of this study unsuitable for extrapolation to populations in other countries or continents.

## Conclusions

After adjusting for multiple covariates, we found a significant positive linear correlation between SII and sarcopenia, indicating that a higher SII can increase the risk of sarcopenia in people aged 18–59 in the United States. The quartile 4 group had a higher risk of sarcopenia than the quartile 1 group. Given the predictive value of SII for the risk of sarcopenia, the findings of this study will be beneficial in promoting the use of SII alone or in combination with other tools for risk screening in communities or large populations.

## Data Availability

The datasets generated and analyzed in the current study are available at NHANES website: https://www.cdc.gov/nchs/nhanes/index.htm.

## Abbreviations

SII: Systemic immune-inflammation index
NHANES: National Health and Nutrition Examination Survey
OR: Odds ratio
CI: Confidence interval
EWGSOP: European Working Group on Sarcopenia in Older People
IWGS: International Working Group on Sarcopenia
AWGS: Asian Working Group for Sarcopenia
FNIH: Foundation for the National Institutes
DXA: Dual-energy X-ray absorptiometry
SARC-F: Sarcopenia-five
SARC-CalF: SARC-F combined with calf circumference
BMI: Body mass index
SARC-F + EBM: SARC-F adding elderly and body mass index information scale
CDC: Centers for Disease Control and Prevention
ASM: Appendicular skeletal muscle mass
CKD: Chronic kidney disease
COPD: Chronic obstructive pulmonary disease
CHD: Coronary heart disease
IGF-1: Insulin-like growth factor-1
IL-6: Interleukin-6
CRP: C-reactive protein
TNF-α: Tumor necrosis factor α
GH: Growth hormone
IFG: Impaired fasting glucose
IGT: Impaired glucose tolerance
